# Supplementary material for: Prognostic factors of cytomegalovirus retinitis after hematopoietic stem cell transplantation
Source: PLoS One. 2020 Sep 2;15(9):e0238257. doi: 10.1371/journal.pone.0238257 (PMC7467317; doi:10.1371/journal.pone.0238257)
Supplement: Attachment — Submitted filename: PONE-D-20-13198.docx [file pone.0238257.s002.docx]

The paper by Young-Hoon Park *et al.* presents a retrospective study where the presence of cytomegalovirus retinitis after hematopoietic stem cell transplantation was analysed in more than 4000 patients. The paper is well written and the data well documented.

Minor:

- The title does not reflect the main conclusion of the study.

- the introduction is more focused on the relation between cytomegalovirus retinitis and AIDS than on hematopoietic stem cell transplantation. This needs to be corrected to better introduce the aim of the study.

- Table 5 is quite complex making it hard to read.

Major:

- The authors should consider new scientific pieces of evidence. Please note that on the references there is only one paper from 2019 and one from 2017 being all the others previous to 2017.
